# Microglia Fighting for Neurological and Mental Health: On the Central Nervous System Frontline of COVID-19 Pandemic

**DOI:** 10.3389/fncel.2021.647378

**Published:** 2021-02-18

**Authors:** Elisa Gonçalves de Andrade, Eva Šimončičová, Micaël Carrier, Haley A. Vecchiarelli, Marie-Ève Robert, Marie-Ève Tremblay

**Affiliations:** ^1^Division of Medical Science, University of Victoria, Victoria, BC, Canada; ^2^Axe Neurosciences, Centre de Recherche du CHU de Québec, Université de Laval, Québec City, QC, Canada; ^3^Neurology and Neurosurgery Department, McGill University, Montréal, QC, Canada; ^4^Department of Molecular Medicine, Université de Laval, Québec City, QC, Canada; ^5^Department of Biochemistry and Molecular Biology, University of British Columbia, Vancouver, BC, Canada

**Keywords:** microglia, COVID-19, SARS-CoV-2, central nervous system, cytokines, hypoxia, neurological manifestations, psychosocial stress

## Abstract

Coronavirus disease 2019 (COVID-19) is marked by cardio-respiratory alterations, with increasing reports also indicating neurological and psychiatric symptoms in infected individuals. During COVID-19 pathology, the central nervous system (CNS) is possibly affected by direct severe acute respiratory syndrome coronavirus 2 (SARS-CoV-2) invasion, exaggerated systemic inflammatory responses, or hypoxia. Psychosocial stress imposed by the pandemic further affects the CNS of COVID-19 patients, but also the non-infected population, potentially contributing to the emergence or exacerbation of various neurological or mental health disorders. Microglia are central players of the CNS homeostasis maintenance and inflammatory response that exert their crucial functions in coordination with other CNS cells. During homeostatic challenges to the brain parenchyma, microglia modify their density, morphology, and molecular signature, resulting in the adjustment of their functions. In this review, we discuss how microglia may be involved in the neuroprotective and neurotoxic responses against CNS insults deriving from COVID-19. We examine how these responses may explain, at least partially, the neurological and psychiatric manifestations reported in COVID-19 patients and the general population. Furthermore, we consider how microglia might contribute to increased CNS vulnerability in certain groups, such as aged individuals and people with pre-existing conditions.

## Introduction

At the beginning of the pandemic, it was thought that the coronavirus disease 2019 (COVID-19), caused by severe acute respiratory syndrome coronavirus 2 (SARS-CoV-2) infection, affected only the respiratory system (Wang et al., 2020b). However, increasing reports of olfactory and taste symptoms in infected patients suggested possible central nervous system (CNS) damage (Agyeman et al., 2020). As the scenario evolved, further reports of neurological manifestations in COVID-19 appeared, such as encephalopathies, cerebrovascular disease, as well as psychiatric symptoms of depression or anxiety (Gautier and Ravussin, 2020; Giacomelli et al., 2020; Mao et al., 2020; Poyiadji et al., 2020; Taquet et al., 2020; Wang et al., 2020b). At the same time, the pandemic broadly imposes a high degree of psychosocial stress (Brooks et al., 2020; McGinty et al., 2020; Pierce et al., 2020), a strong predictor of mental health disorders (Maes et al., 1998; Schneiderman et al., 2005), on the general population. Similar coronaviruses (CoVs), such as severe acute respiratory syndrome coronavirus 1 (SARS-CoV-1) and Middle East respiratory syndrome coronavirus (MERS-CoV) were also recently associated with psychiatric and neurological disorders, with a prevalence of 0.09% for SARS-CoV-1 and 0.36% for MERS-CoV (Ellul et al., 2020). Despite a seemingly low proportion, given the large number of reported COVID-19 cases [78,383,527, as of December 26th 2020 WHO Coronavirus Disease (COVID-19) Dashboard (2020)], this may indicate approximately 70,545 people impacted, if a similar ratio is observed for the SARS-CoV-2 (Ellul et al., 2020).

The cause of these diverse manifestations remains elusive. To help stimulate and orient further research on the consequences of COVID-19 on neurological and mental health, in this review, we discuss several putative origins, which include SARS-CoV-2 infection in the CNS, hypoxia-derived injuries in the brain, and the excessive circulation of inflammatory factors in COVID-19 affected individuals. Acting synergistically or not, we suggest how these factors trigger protective and neurotoxic responses by microglia, the resident innate immune cells of the CNS, along with their possible connections to the neurological and psychiatric manifestations encountered upon SARS-CoV-2 infection. Lastly, we consider the burden of the psychosocial stress imposed by the pandemic in both COVID-19 affected individuals and the general population. We cover how, even in the absence of infection, microglia might respond to stress and severely impact the mental health of vulnerable groups.

## Microglia Mediate How the CNS Is Affected by SARS-CoV-2 Infection

### The CNS Is Affected by Direct SARS-CoV-2 Infection

**FIGURE 1:**
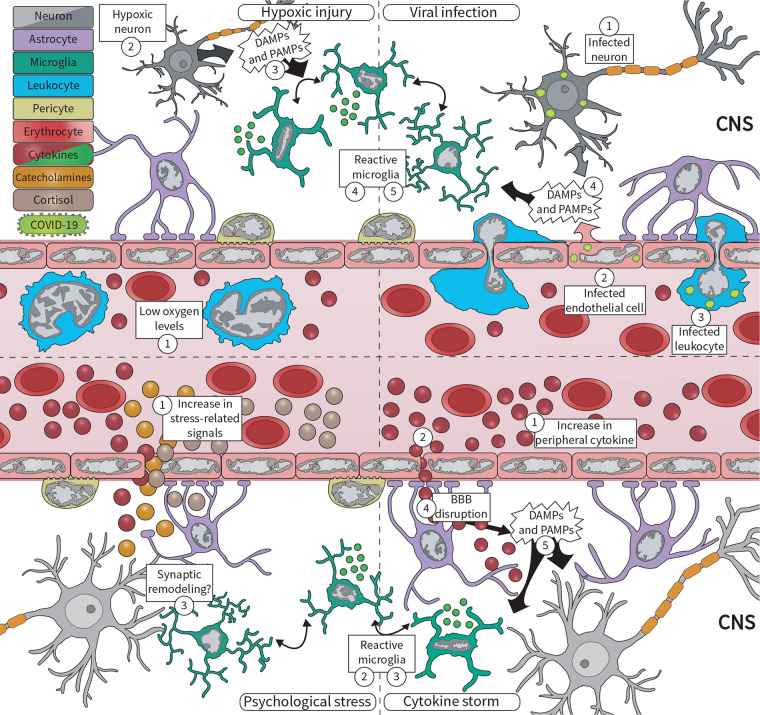
During the COVID-19 pandemic, microglia respond to various central nervous system (CNS) insults, including viral infection, hypoxic injury, excessive circulating cytokines, and psychosocial stress. (Upper panel) Severe acute respiratory syndrome coronavirus 2 (SARS-CoV-2) can enter the brain via (1) peripherally infected neurons, or via (2) infected microvascular brain endothelial cells, and (3) infected infiltrating leukocytes. Infected cells, in turn, (4) release damage-associated molecular patterns (DAMPs) and pathogen-associated molecular patterns (PAMPs) that are sensed by microglia, which respond by (5) becoming reactive. At the same time, COVID-19 pathology contributes to (1) low oxygen levels, likely driving hypoxic injuries in the brain. Neurons are extremely sensitive to oxygen deficits, becoming (2) quickly damaged, and (3) releasing DAMPs and PAMPs. This is sensed by (4) microglia, which respond by changing their morphology, molecular signature, and cytokine release. (Lower panel) COVID-19 is also associated with an exacerbated inflammatory response, marked by increased levels of (1) circulating cytokines, i.e., cytokine storm. These cytokines signal to the brain via different pathways, for example, (2) signaling through the blood–brain barrier (BBB), being quickly sensed by (3) microglia. Furthermore, excessive cytokines may drive (4) BBB disruption, which increases not only cytokine levels in the brain but also of (5) DAMPs and PAMPs associated with the systemic viral infection. Lastly, psychosocial stress drives the production of cortisol, catecholamines, and cytokines, released into the (1) circulation. Microglia can respond to all of these factors by becoming (2) reactive, likely driving dysfunctional (3) synaptic remodeling.

The CNS-associated manifestations of COVID-19 could, in part, result from SARS-CoV-2 infection in the brain. Two main routes for CNS invasion, based on either (i) neuronal or (ii) hematogenous transport (Figure 1), were proposed in the literature.

#### Neuronal Route

The neuronal route relies on retrograde axonal transport from infected peripheral nerves, such as the olfactory or vagus nerves (Desforges et al., 2019; DosSantos et al., 2020). Many SARS-CoV-2 related viruses, including other CoVs, use olfactory nerve fibers to enter the CNS (Koyuncu et al., 2013). In transgenic mice expressing the human angiotensin I converting enzyme 2 (ACE2) protein, a high-affinity receptor for SARS-CoVs, intranasal SARS-CoV-1 administration resulted in olfactory nerve infection, progressively spreading to the olfactory bulb, cerebral cortex, basal ganglia, and midbrain (Netland et al., 2008). Similarly, in a patient who died from a severe respiratory failure associated with COVID-19, SARS-CoV-2 viral particles were detected in the olfactory nerve, gyrus rectus, and brainstem (Bulfamante et al., 2020). Likewise, immunohistochemical analysis revealed SARS-CoV-2 proteins in the cranial nerves originating from the lower *medulla oblongata* and a high degree of reactive glial cells, i.e., gliosis, in the olfactory bulb of autopsied COVID-19 patients (Matschke et al., 2020). Infection of the olfactory system is also consistent with the frequent loss of smell and increased magnetic resonance imaging (MRI) signal measured in the olfactory cortex of patients (Lu et al., 2020; Politi et al., 2020). It is important to note, however, that it remains unclear whether ACE2 and transmembrane serine protease 2 (TMPRSS2), another SARS-CoV-2 receptor, are expressed specifically by neurons of the olfactory system (Brann et al., 2020; DosSantos et al., 2020).

Abundant ACE2 expression in small intestine endothelial cells (ECs) (Hamming et al., 2004), and the incidence of gastrointestinal symptoms in patients with COVID-19 (Chen et al., 2020a; Wang et al., 2020b), also prompted a putative neuronal route involving the enteric nervous system. Indeed, this strategy has been proposed for several neurotropic viruses, including CoVs (DosSantos et al., 2020). For instance, intragastric inoculation with MERS-CoV in transgenic mice expressing human dipeptidyl peptidase 4 (DPP4) protein, the MERS-CoV receptor, led to enterocyte, lung, and brain infection (Zhou et al., 2017). The path used by these viruses to reach the CNS is unclear but they could follow the intestinal nervous plexuses and vagus nerve, reaching the brainstem through axonal transport (DosSantos et al., 2020). While the presence of SARS-CoV-2 in these nerves remains obscure, human small intestinal organoids have shown to be productively infected by the novel coronavirus (Lamers et al., 2020). Notably, enteric glial cells recognize and fight viruses, coordinating both innate and adaptive antiviral responses that are connected to neurological impairment, for example, via the release of the pro-inflammatory cytokine interleukin (IL) 6 (Esposito et al., 2020).

#### Hematogenous Route

Yet, SARS-CoV-2 could also use a hematogenous route, i.e., via the bloodstream, infecting brain microvascular ECs or leukocytes crossing the blood–brain barrier (BBB) to reach the CNS (Figure 1; Desforges et al., 2019). The BBB is composed of a monolayer of ECs joined by tight junctions, and it is covered by pericytes, as well as astrocytic (Abbott et al., 2006), and microglial endfeet (Joost et al., 2019). This structure limits almost all transcellular and paracellular transport from the blood to the brain parenchyma, protecting the CNS from pathogens and toxins (Abbott et al., 2006). *In vitro* human and rodent studies have suggested all components of the BBB can be infected by viruses (Chen and Li, 2020). Correspondingly, ACE2 protein is broadly present in ECs of post-mortem human brains (Hamming et al., 2004; Buzhdygan et al., 2020). Furthermore, budding of SARS-CoV-2 viral particles has been observed in the brain ECs of a deceased COVID-19 patient (Paniz-Mondolfi et al., 2020). In parallel, SARS-CoV-2 infiltration through the brain vasculature could be facilitated by BBB disruption (Alquisiras-Burgos et al., 2020). Endothelial infection increasing BBB permeability was associated with the mouse CoV, i.e., neurotropic mouse hepatitis virus (MHV), through down-regulation of proteins forming the tight junctions between brain ECs, such as tight junction protein 1 (TJP1), cadherin 5, and occludin (Bleau et al., 2015). SARS-CoV-2 spike proteins (subunits 1 and 2) also increased permeability of an *in vitro* 3D BBB model constituted of primary human brain ECs, as assessed by enhanced dextran perfusion and decreased TJP1 immunostaining (Buzhdygan et al., 2020). In brain ECs, this effect was accompanied by higher messenger RNA (mRNA) expression of the (i) cytokines *IL6* and *IL1B*, (ii) matrix metallopeptidases (*MMPs*) *2, 3, 9*, and *12*, involved in remodeling of the extracellular matrix; (iii) leukocyte chemotaxis factors, such as C-C motif chemokine ligand 5 (*CCL5*) and C-X-C motif chemokine ligand 10 (*CXCL10*); as well as protein expression of (iv) cell adhesion molecules, including intracellular adhesion molecule 1 and vascular cell adhesion protein 1 (Buzhdygan et al., 2020).

To reach the CNS, the SARS-CoV-2 could likewise use the blood-cerebrospinal fluid (CSF) barrier, a more permeable barrier compared to the BBB, formed by a single layer of ECs of the choroid plexus (Pellegrini et al., 2020). SARS-CoV-2 receptors, including ACE2 and TMPRSS2, were detected in human choroid plexus organoids via transcriptomic and immunoblotting analysis (Pellegrini et al., 2020). Pellegrini et al. (2020) also observed that SARS-CoV-2 clinical isolate infected choroid plexus but not neuronal organoids; and this infection led to a disruption of tight junctions, labeled by claudin 5 (CLDN5), and an overall breakdown of barrier integrity. Although similar reports are rare, in a case series examining 30 COVID-19 patients with neurologic symptoms, five showed a high CSF-blood albumin ratio, suggesting either BBB or blood-CSF barrier disruption (Neumann et al., 2020). Lastly, instead of using the endothelium, SARS-CoV-2 CNS invasion could occur via infected immune cells that infiltrate the brain. Yet, only macrophages were positive for the virus according to single-cell RNA sequencing (RNA-seq) of bronchoalveolar lavage samples from COVID-19 patients (Bost et al., 2020). Of note, this finding could be due to phagocytosis of the viral components or infected cells, and not necessarily to viral propagation. Additionally, thus far, autopsy series of deceased COVID-19 patients did not show marked peripheral immune cell infiltration in the brain (Iadecola et al., 2020), and dissemination of SARS-CoV-2 into the blood has been described in inconsistent proportions, ranging from 1 (Wang et al., 2020b) to 41% (Zheng et al., 2020).

#### Evidence of SARS-CoV-2 Brain Infection

The path used by SARS-CoV-2 to invade the CNS remains puzzling, along with the fate of the virus once it reaches the brain. On one hand, human neural organoids seem to be limited in their ability to support SARS-CoV-2 replication (Pellegrini et al., 2020; Ramani et al., 2020). On the other, dopaminergic neurons derived from human pluripotent cells were found to be susceptible to SARS-CoV-2, but not cortical neurons, brain ECs, macrophages, or microglia (Yang et al., 2020a); despite the RNA expression of SARS-CoV-2 receptors in several CNS cell types. According to human brain single-cell RNA-seq, *ACE2* is expressed in many neuronal subtypes, astrocytes, and oligodendrocytes (Matschke et al., 2020). Expression of *TMPRSS2* and *TMPRSS4* was most elevated in neurons, whereas cathepsin L (*CTSL*), necessary for viral entry and replication, was highest in microglia (Matschke et al., 2020). However, the protein expression of these receptors in the CNS still needs to be investigated, along with SARS-CoV-2 ability to replicate in the different brain cell types *in vivo*.

Notably, SARS-CoV-2 CNS invasion is also supported by viral detection in both the CSF and brain of COVID-19 patients (Bulfamante et al., 2020; Domingues et al., 2020; Huang et al., 2020; Kremer et al., 2020; Matschke et al., 2020; Moriguchi et al., 2020; Paniz-Mondolfi et al., 2020; Puelles et al., 2020; Solomon et al., 2020; Virhammar et al., 2020). It is clear, though, that presence of SARS-CoV-2 in CSF is rare (Domingues et al., 2020; Huang et al., 2020; Kremer et al., 2020; Moriguchi et al., 2020; Virhammar et al., 2020). For example, in a study with 31 CSF human samples, only one was positive for SARS-CoV-2 via quantitative reverse transcription polymerase chain reaction (RT-qPCR) (targeting the RNA-dependent RNA polymerase sequence), despite increased CSF markers of inflammation, such as high protein count or elevated immunoglobulin G in all of them (Kremer et al., 2020). Similarly, several other studies evaluating a total of 30 COVID-19 patients with neurological manifestations did not detect SARS-CoV-2 in the CSF by RT-qPCR (targeting E and nucleocapsid genes) (Al Saiegh et al., 2020; Al-Dalahmah et al., 2020; Alexopoulos et al., 2020; Filatov et al., 2020; Helms et al., 2020; Paniz-Mondolfi et al., 2020; Schaller et al., 2020). At the same time, definite proof of SARS-CoV-2 invasion in the brain via autopsied tissue, while limited, was reported in patients showing severe COVID-19 and a history of previous chronic diseases (Matschke et al., 2020). Using electron microscopy, the first report detected viral particles in brain ECs and frontal lobe neurons of a 74-years old patient with a history of Parkinson’s disease (PD) (Paniz-Mondolfi et al., 2020). SARS-CoV-2 viral particles were also uncovered in the brainstem of another COVID-19 deceased patient (see section “Neuronal Route”) (Bulfamante et al., 2020). Later on, five out of 18 (Solomon et al., 2020) and eight out of 22 (Puelles et al., 2020) post-mortem human brains were shown to be positive for SARS-CoV-2 RNA (E and nucleocapsid genes) but negative for immunohistochemical analysis of nucleocapsid (Solomon et al., 2020) and spike (Puelles et al., 2020) proteins. Yet, in the largest study published so far, 21 out of 40 affected individuals displayed both SARS-CoV-2 RNA and proteins in the brain, based on RT-qPCR (E gene) and immunohistochemistry (nucleocapsid and spike proteins) (Matschke et al., 2020).

Inconsistent SARS-CoV-2 detection in the CNS and CSF contributes to the great uncertainty around the novel coronavirus’ level of neurotropism. For instance, in one study, the virus was undetectable in CSF despite being present in the patient’s brain (Paniz-Mondolfi et al., 2020). Similarly, brain samples of eight patients were negative for viral RNA yet immunopositive for the spike or nucleocapsid proteins in another study (Matschke et al., 2020). Even more intriguingly, SARS-CoV-2 was reported in the CSF of one patient without respiratory symptoms (Huang et al., 2020). Conflicting results for RT-qPCR SARS-CoV-2 detection in CSF, and between RT-qPCR and immunohistochemistry in the brain, reveal a need for more cohesion in tests to properly estimate the prevalence of brain infection (Murta et al., 2020). Similarly, since the putative routes and outcomes for CNS invasion are drawn mostly from correlative data, it is imperative to directly investigate what pathway(s) are used by SARS-CoV-2 and if productive infections can be generated in the brain. In the event of SARS-CoV-2 brain infection, the fate of CNS function relies on antiviral defenses; and microglia, as the resident innate immune cells of the brain, could hinder viral spread, as discussed below.

### Putative Responses of Microglia to SARS-CoV-2 CNS Infection

Microglia, contrary to all the other CNS cells, arise from erythro-myeloid progenitors in the fetal yolk sac and are present in the brain during embryonic development, self-renewing throughout life (Ajami et al., 2007; Ginhoux et al., 2010; Tay et al., 2017b). These cells are central players in the inflammatory response of the brain, i.e., neuroinflammation, in most if not all physical, infectious, psychiatric, or neurodegenerative-related insults to the CNS. Microglia are also essential for brain development, plasticity, and homeostasis, contributing to (i) the turnover (both elimination and survival) of neuronal precursors and neurons, (ii) oligodendrocyte progenitor cell maturation, (iii) neuronal wiring, (iv) synaptic maturation, activity, and plasticity, (v) myelination, as well as (vi) BBB integrity, and (vii) blood flow regulation (Tay et al., 2017b). Considering their diverse role in the brain’s immune and physiological functions, we hypothesize that microglia respond to direct SARS-CoV-2 CNS infection and changes in the CNS imposed by systemic COVID-19 response. In addition to microglia, peripheral immune cells, derived from the bone marrow, and able to enter the CNS from the circulation throughout life, such as T lymphocytes and macrophages, could assist microglia with the response to COVID-19 (Zhao et al., 2014; Wheeler et al., 2018; Klein et al., 2019; Theret et al., 2019; Mangale et al., 2020).

Innate immunity is fundamental for viral clearance in the CNS. This response is initiated by sensing pathogen-associated molecular patterns (PAMPs) and damage-associated molecular patterns (DAMPs) via pattern recognition receptors (PRRs). SARS-CoV-2 is a single-stranded RNA (ssRNA) virus that, similarly to SARS-CoV-1 and MERS-CoV, is thought to produce double-stranded RNA (dsRNA) molecules during replication. PRRs, such as Toll-like receptors (TLRs), or the cytoplasmic retinoic acid-inducible gene I-like receptors (RLRs), recognize these PAMPs; TLR3 and RLRs, including the DEAD/H box helicase 58 and the interferon induced with helicase C domain 1, recognize dsRNAs, whereas TLR7 and TLR8 recognize ssRNAs (de Wit et al., 2016; Carty et al., 2021). These PRRs are expressed mainly by microglia (Kumar, 2019; Carty et al., 2021), but also by neurons, astrocytes, pericytes, and brain ECs (Klein et al., 2019; Chen and Li, 2020). Activation of TLRs and RLRs leads to the expression of type I interferons (IFN) via the interferon regulatory factor 3/7, and of pro-inflammatory cytokines via the nuclear factor kappa B (NFKB), thereby initiating an antiviral cascade (Carty et al., 2021).

Upon binding of cytokines, DAMPs, and PAMPs to their appropriate receptors, microglia initiate an inflammatory response (Klein et al., 2019), changing their gene expression, morphology, and function in a process known as “reactivity” or “microgliosis” (though previously termed “activation”) (Hoogland et al., 2015; Savage et al., 2019). Among their adjustments, in rodents, microglia (similar to peripheral macrophages in the brain) can up-regulate protein expression of the ionized calcium-binding adapter molecule 1 (IBA1), the major histocompatibility complex class II (MHCII), and the phagolysosomal marker cluster of differentiation (CD) 68 (Jurga et al., 2020). Reactive microglia can proliferate and increase the release of pro-inflammatory cytokines [tumor necrosis factor (TNF), IL1B, IL6, IFNG] as well as reactive oxygen species (ROS) or nitric oxide (NO) which, balanced by concomitant anti-inflammatory cytokine release by microglia, help restore homeostasis (Lund et al., 2006; Block et al., 2007; Cunningham, 2013). Besides, reactive microglia generally adopt different morphologies, for example, shifting to amoeboid shapes with bigger somas and less ramified processes, as opposed to the steady-state, highly branched “surveilling” phenotype (Figure 1; Savage et al., 2019; Tremblay et al., 2020). Accordingly, increased IBA1 immunostaining and less ramified CX3C chemokine receptor (CX3CR1)-green fluorescent protein (GFP)-positive microglial cells were observed in the olfactory bulb of MHV infected mice (Wheeler et al., 2018). Increased IBA1-positive microglial cells were also described in mice expressing the human ACE2 protein, 6 days following intranasal SARS-CoV-1 infection (Netland et al., 2008).

Reactive microglial phenotypes can secrete antiviral factors, such as IFNs and IL6 (Klein et al., 2019). However, other cells can compensate for this release in the absence of normal levels of microglia (Wheeler et al., 2018). This has been shown by manipulating signaling through the colony stimulating factor 1 receptor (CSF1R), a receptor tyrosine kinase required for the development, maintenance, and proliferation of microglia (Ginhoux et al., 2010; Erblich et al., 2011; Elmore et al., 2014). Using its antagonist PLX5622 to significantly deplete CSFR1-positive cells, including microglia (but not the resident CD45-high macrophage population that can infiltrate the CNS from the circulation), Wheeler et al. (2018) showed *Ifnb*, *Ifna*, *Il6* mRNA levels were not changed in mouse brains infected with neuro-attenuated rJ2.2 strain of the murine hepatitis virus (JMHV). Similarly, in the case of a neurovirulent strain of MHV, gene set enrichment analysis from single-cell RNA-seq of *ex vivo* CD45-high cells isolated from the brain revealed enrichment of IFN-response genes in peripheral macrophages and dendritic cells of infected versus non-infected PLX5622-treated mice (Mangale et al., 2020). This compensatory mechanism is also supported by *in vitro* studies showing that murine astrocytic cells are important sources of IFNA/B after infection with MHV strain A59 (Savarin and Bergmann, 2018; Lavi and Cong, 2020).

Similarly to other CoVs, SARS-CoV-2 can infect and drive permeability of an *in vitro* human BBB model (Bleau et al., 2015; Buzhdygan et al., 2020). BBB disruption, in turn, can enhance the entry of peripheral immune cells and inflammatory factors in the brain to help fight the infection. Although microglia do not appear to be critical for overall immune cell infiltration (Figure 1), they are crucial for initiating the antiviral responses of peripheral cells. For instance, significant reductions in the microglial population (mediated by PLX5622) early (0 and 6 days) after intracranial JMHV infection severely impacted viral restriction, resulting in an increased viral load and a higher mortality rate of mice (Wheeler et al., 2018). Microglial depletion (i) disrupted antigen-presentation, and consequently CD4-positive T cell and regulatory T cell (Treg) antiviral responses, and (ii) led to immature macrophage infiltration (Wheeler et al., 2018; Mangale et al., 2020). Conversely, peripheral cells seem to be important for microglial regulation. Intravenous administration of Foxp3-positive CD4 regulatory T cells recognizing the viral MHV M133 epitope 24 h before MHV infection decreased microglial MHCII protein expression and limited T helper type 1 (Th1) cell response, improving survival of mice (Zhao et al., 2014). The close interactions between brain resident immune cells and virus-specific T cells are also supported by significant gene expression changes in pathways related to (i) antigen presentation, (ii) crosstalk between the innate and adaptive immune systems, (iii) IFN signaling, (iv) IFN-regulatory factors, (v) PRRs, and (vi) chemokines in microglia from MHV infected mice compared to controls (Wheeler et al., 2018).

While coordinating the antiviral response, microglia can themselves become infected. This has been shown for CoVs including the human coronavirus OC43, as well as the murine strains MHV and JHMV (Bonavia et al., 1997; Arbour et al., 1999; Nakagaki et al., 2005; Das Sarma, 2014; Lavi and Cong, 2020). Although, except for MHV, this infection of microglia was observed only at low levels, and mostly in immortalized cell lines from humans and mice, which do not necessarily correlate with *in vivo* conditions (Bonavia et al., 1997; Arbour et al., 1999; Nakagaki et al., 2005; Das Sarma, 2014; Lavi and Cong, 2020). Evidence from different viral families suggests that upon direct infection, microglia show signs of reactivity. For example, in primary microglial cultures from rats, the Japanese encephalitis virus (JEV) drives a change from a process-bearing morphology to an amoeboid and CD68-positive cell, characteristic of a phagocytic phenotype (Chen et al., 2010). Remarkably, this morphological shift seemed to be induced by soluble components released by infected cells and not by active viral replication (Chen et al., 2010). Initial results with human microglial cells derived from pluripotent cells argue these cells are not susceptible to SARS-CoV-2, despite ACE2 protein and *CTSL* mRNA expression (Matschke et al., 2020; Yang et al., 2020a) [though further research in humans is necessary to confirm this finding].

Among the reports evaluating microglial markers in post-mortem brain tissue from COVID-19 cases, one detected SARS-CoV-2 brain invasion in 21 out of 40 patients (see also section “Evidence of SARS-CoV-2 Brain Infection”) (Matschke et al., 2020). Matschke et al. (2020) observed diffuse staining for IBA1 and the microglial enriched marker transmembrane protein 119 (TMEM119), with occasional microglial nodules, i.e., microglial clusters, among the brainstem and cerebellum in 29 out of 32 tested COVID-19 patients. In the subpial and subependymal regions, IBA1-positive microglia were frequently seen surrounded by CD8-positive T cells, and strongly expressed CD68, as well as the human leukocyte antigen DR isotype (HLA-DR, an MHCII receptor) (Matschke et al., 2020). However, it is not clear if the same patients who had detectable brain levels of SARS-CoV-2 were also those that showed an increase in these microglial markers (Matschke et al., 2020). Up-regulated microglial protein expression of IBA1, CD68, TMEM119, as well as HLA-DR, and their proximity to T cells, could indicate active phagocytosis of antigens to drive T cell activation and CNS infiltration following viral infection, as observed previously with other CoVs (Wheeler et al., 2018; Mangale et al., 2020). At the same time, the changes in microglia detected in this work could stem from or be potentiated by a response to the systemic COVID-19, which on its own is associated, for example, with hypoxic brain damage, as will be discussed next.

## Microglia Could Determine How the CNS Is Affected by COVID-19 Pathology

### Microglia Respond to COVID-19-Associated Hypoxia

Although 81% of SARS-CoV-2 infected individuals present mild pneumonia, severe disease-associated hypoxia is observed in around 14%, critical disease in 5%, and death in 2.3% of patients (Wu and McGoogan, 2020). In severe cases, the most expressive pathological consequences of COVID-19 include massive alveolar damage, heart failure, coagulopathy, and cerebrovascular disease, including ischemic events (Aggarwal et al., 2020; Carsana et al., 2020; Chen et al., 2020b; Helms et al., 2020; Klok et al., 2020; Mao et al., 2020; Oxley et al., 2020). All factored in, these systemic changes likely drive hypoxic brain injury (Figure 1; Iadecola et al., 2020). Consistently, COVID-19 patients often present symptoms related to CNS hypoxia, such as headache, drowsiness, and coma, as well as brain lesions (Chen et al., 2020a; Giacomelli et al., 2020; Mao et al., 2020; Pezzini and Padovani, 2020; Wu et al., 2020a). In brain autopsies of COVID-19 patients, widespread microthrombi and patches of infarction have also been detected, together with neuronal damage in the cerebral cortex, hippocampus, and cerebellum, i.e., brain areas highly vulnerable to hypoxia (Kantonen et al., 2020; Kashani et al., 2020; Solomon et al., 2020) [although these injuries could also be present before COVID-19].

Ischemic-hypoxic brain injury is partially produced by a switch to anaerobic metabolism in brain cells, leading to (i) accumulation of lactic acid in the parenchyma, (ii) oxidative stress, (iii) BBB dysfunction, (iv) cerebral vasodilation, (v) swelling of neurons, (vi) obstruction of blood flow, (vii) inflammation, and (viii) cell death (Fumagalli et al., 2015; Wu et al., 2020b). Microglia rely on ATP to reorganize their cytoskeleton and monitor the brain parenchyma (Atkinson et al., 2004; Davalos et al., 2005; Masuda et al., 2011; Gimeno-Bayón et al., 2014), making them highly sensitive to energy deficits (Fumagalli et al., 2015). During COVID-19-associated hypoxia, it is therefore likely that changes in the ATP micro gradient are sensed by microglial purinergic P2X and P2Y receptors, promoting their migration to the injury site, phagocytosis, and proliferation, as observed in hypoxic injuries of rodents and humans (Davalos et al., 2005; Melani et al., 2006; Wixey et al., 2009; Webster et al., 2013; Fukumoto et al., 2019).

Hypoxia also triggers rodent microglial transcription factors (e.g., hypoxia inducible factor 1, alpha subunit and NFKB), and micro RNAs (miR-s) (e.g., miR-146a, and miR181a/c), both *in vitro* and *in vivo*; resulting in the release of TNFA, ROS, IL1B, and IL18, which can help clear cellular debris and resolve the injury (Zhang et al., 2012; Kong et al., 2014; Kiernan et al., 2016; Mukandala et al., 2016; Jiang et al., 2020; Yang et al., 2020b). In response to ischemia-hypoxia, rodent microglia likewise transform morphologically (see Figure 1; Masuda et al., 2011). Acutely, in the cerebral cortex, mouse microglia expand their processes to reach the hypoxic area (Davalos et al., 2005; Hines et al., 2009), but in larger or more sustained injuries, microglia can also adopt an amoeboid morphology (as observed during viral infections) among the hippocampus and neocortex of rodents (Raivich et al., 1999; Stence et al., 2001; Kurpius et al., 2007; Masuda et al., 2011). Whether microglia could react similarly during COVID-19 pathology awaits further research.

Reactive microglia can perform neurotoxic and neuroprotective roles after hypoxic-ischemic injury. Disrupting fractalkine-mediated neuron-microglia signaling, by knocking out CX3CR1 in mice after middle cerebral artery occlusion (MCAO)-induced ischemia, decreased the proliferation of microglia, release of inflammatory molecules, and infiltration of macrophages, culminating in a smaller ischemic lesion (Fumagalli et al., 2013; Tang et al., 2014). Contrastingly, combining PLX3397 driven microglial depletion with high-resolution two-photon calcium imaging *in vivo*, Szalay et al. (2016) showed 24 h after MCAO that microglial depletion impaired neuronal calcium responses and network activity, while increasing intracellular calcium levels, ultimately leading to exacerbated neuronal damage in mice. Yet, peripheral administration of minocycline, a tetracycline antibiotic that non-specifically normalizes microglial phagocytosis and release of pro-inflammatory mediators (Tikka and Koistinaho, 2001; Tikka et al., 2001; Hanisch and Kettenmann, 2007), attenuated neuronal death in rodent models of ischemia (Yrjänheikki et al., 1998, 1999). Thus, similar to what was previously discussed in section “Putative Responses of Microglia to SARS-CoV-2 CNS Infection” (regarding depleting microglia during CNS viral infection), while completely disrupting microglial function can worsen injury recovery, down-regulating their inflammatory response can be protective. Of note, minocycline treatment has been proposed for COVID-19 (Oliveira et al., 2020).

Microglial nodules and elevated immunostaining for microglial reactivity markers (e.g., IBA1, HLA-DR, and CD68) were detected in numerous brain autopsies of COVID-19 patients also showing recent and older hypoxic/ischemic injuries (Al-Dalahmah et al., 2020; Kantonen et al., 2020; Matschke et al., 2020), accompanied by marked neuronal loss among the cerebral cortex, hippocampus, medulla, and cerebellar Purkinje cell layer (Al-Dalahmah et al., 2020; Solomon et al., 2020). In a patient showing severe global hypoxic changes with hypereosinophilic, shrunken neurons, microglial CD68-positive nodules were surrounding the injured cells in the inferior olives and dentate nuclei, possibly indicating active phagocytic removal (Al-Dalahmah et al., 2020). However, at this point, it is difficult to evaluate whether the observed changes in the resident immune cells stem from the hypoxic injuries associated with the COVID-19. Reactive microglia may arise due to other systemic changes associated with SARS-CoV-2 infection, including the exacerbated peripheral immune response (Al-Dalahmah et al., 2020), as we consider next.

### Microglia Respond to the Systemic Inflammatory Response in COVID-19

A well-coordinated immune response represents the first line of defense against viral infection. Yet, if exacerbated, this response may become detrimental, at both the viral entry site and systemic levels. As previously reported for SARS-CoV-1 and MERS-CoV (Channappanavar and Perlman, 2017), a maladaptive innate and adaptive immune response is a hallmark of COVID-19 pathology (Qin et al., 2020). Its most apparent characteristic is an ostensible hyper-inflammation, popularly termed “cytokine storm” prevalent in severely affected patients and significantly contributing to multi-organ failure (Bhaskar et al., 2020; Moore and June, 2020; Ruan et al., 2020). The putative origin of this phenomenon lies in a three-step process, consisting of an initial immune activation, a secondary delayed but possibly prolonged antiviral IFN-mediated response, and an uncontrolled monocyte-macrophage-dendritic cell hyperactivation as well as tissue infiltration (Channappanavar and Perlman, 2017; Merad and Martin, 2020; Moore and June, 2020). Such a process may induce vascular and organ damage on the cellular level and contribute to overall inefficient handling of viral load due to sub-optimal T- and B-cell responses (Channappanavar and Perlman, 2017). Accordingly, a reduction in serum lymphocytes is observed in a subset of COVID-19 patients (Bhaskar et al., 2020; Mathew et al., 2020). Within the cytokine storm framework, elevated circulating levels of an extensive variety of pro- and anti-inflammatory cytokines and chemokines (e.g., IL1; IL1R1; IL6; IL8; IL7; IL10; IL12; IFNG; Transforming growth factor b; CCL2; CXCL10; CXCL9; CX3CL10; CCR1) as well as non-cytokine markers (C-reactive protein, CRP; CSF2; D-dimer; ferritin), were reported in COVID-19 patients up to this point (Heneka et al., 2020; Mathew et al., 2020; Merad and Martin, 2020; Ruan et al., 2020). It should be noted, however, that the profile of this systemic inflammatory response, in terms of individual markers, varied between cohorts, which could either be a consequence of specific profiling of these initial studies or be reflective of a strong individual-specific nature of this response.

Systemic cytokines communicate with the CNS via several pathways, including (i) migration through leaky regions of the BBB, such as the circumventricular organs; (ii) active transport by cytokine-specific transporters expressed on the brain ECs; (iii) activation of brain ECs leading to the production of second messengers; (iv) transmission of the signal via the vagus nerve, and (v) entry via peripherally activated immune cells (Capuron and Miller, 2011). In the CNS, controlled levels of these soluble substances including IL1B, TNF, and IL6 are necessary for the proper function of both neurons and glial cells (Camacho-Arroyo et al., 2009; Borsini et al., 2015). During infection, systemic cytokines stimulate neuroendocrine responses via the activation of the hypothalamus-pituitary-adrenal (HPA) axis and the elevation of the core body temperature, ultimately promoting disease-specific behavioral patterns (i.e., sickness behavior), such as lethargy and reduced appetite (Szelényi, 2001; Dantzer, 2018). In the long-term, however, elevated cytokine levels in the brain parenchyma (Figure 1) serve as a mediator of neurotoxic and neurodegenerative pathology across various disease conditions (Szelényi, 2001; Camacho-Arroyo et al., 2009).

In the context of COVID-19, pathological excess of cytokines may lead to vascular remodeling and BBB leakage, increasing the entry of DAMPs and PAMPs associated with the peripheral viral infection (Figure 1), which could be especially risky in individuals whose BBB is already impaired due to pre-existing disease conditions (Varatharaj and Galea, 2017). This is in line with MRI-detected BBB-related abnormalities present in COVID-19 patients, such as the frequent occurrence of microbleeds, especially in white matter regions (Fitsiori et al., 2020; Kremer et al., 2020; Radmanesh et al., 2020). This BBB permeability may be primarily driven by (i) systemic events, such as tight junction alterations observed in diabetes (Hawkins et al., 2007), which is a risk factor for COVID-19 complications (Guo et al., 2020); or (ii) via direct viral infection of brain ECs (Buzhdygan et al., 2020); and, possibly, (iii) hypercoagulation and associated microthrombi formation, as was previously observed in COVID-19 patients (Dolhnikoff et al., 2020). On the other hand, viral entry and the neuroinflammatory response may even precede BBB impairment, as it has been stipulated for JEV-infected mice (Li et al., 2015; Pezzini and Padovani, 2020).

Considering their key roles in brain homeostasis and neuroinflammation, microglia are particularly sensitive to environmental perturbations (see section “Putative Responses of Microglia to SARS-CoV-2 CNS Infection”) (Hanisch and Kettenmann, 2007; Hoogland et al., 2015). Thus, the increase in circulating cytokines during COVID-19 may induce or exacerbate microglial reactivity (Figure 1; Tremblay et al., 2020), likely worsening the direct viral or hypoxic injuries possibly present in the patients. The long-term duration of the systemic cytokine storm could contribute to a chronically reactive microglial state, adversely impacting the survival of neurons and maintenance of synaptic connections via inflammatory signaling, phagocytosis, and oxidative stress (Savage et al., 2019). Accordingly, in a patient with increased CSF and serum levels of IL6, IL8, and TNF, the microglial marker triggering receptor expressed on myeloid cells 2 (TREM2), associated with neurodegenerative diseases and phagocytosis, was also increased in the CSF, indicating an active inflammatory process in the spinal cord meningeal space (Pilotto et al., 2020a,b). Likewise, the expansion of inflammatory-oriented microglial clusters marked by increased intensity of IBA1, HLA-DR, and CD68 immunoreactivity and increased density of microglial nodules found in post-mortem brains of COVID-19 patients would be in line with this ongoing microgliosis (Mukandala et al., 2016; Matschke et al., 2020). Microgliosis, further possibly accompanied by astrogliosis and brain infiltration of peripheral T-lymphocytes, was mostly localized within the cerebellum and the brainstem of COVID-19 patients (Mukandala et al., 2016; Matschke et al., 2020). Similarly, in mice, the brainstem is among the brain areas most affected by SARS-CoV-1 and MERS-CoV infection (McCray et al., 2007; Netland et al., 2008; DosSantos et al., 2020). As the brainstem is responsible for vital brain functions (e.g., regulation of cardiac and respiratory functions, sleep cycles, and consciousness), this may be of relevance for the development of breathing difficulties associated with COVID-19 (Nouri-Vaskeh et al., 2020). However, as the cohorts analyzed in the above-mentioned studies are still quite small, further research is necessary to confirm these observations.

## Microglial-Mediated CNS Inflammation May Contribute to the COVID-19 Associated Neurological Manifestations

Although data is still emerging, reports are indicating that presently, approximately 30% of COVID-19 patients who are hospitalized display neurological symptoms (Helms et al., 2020; Mao et al., 2020), which include malaise, headache, and loss of smell (anosmia) and taste (dysgeusia) (Mao et al., 2020), as well as more serious complications, such as ischemic stroke [associated with significantly higher mortality] (Merkler et al., 2020; Yaghi et al., 2020). Drawing on what has been discussed in the previous sections, next we propose how the neurological abnormalities observed in COVID-19 patients could be related to the neuroinflammation resulting from microglial responses to viral, hypoxic, and inflammatory insults.

### Loss of Smell and Taste

Many patients with COVID-19 exhibit symptoms of dysgeusia or anosmia (Eshraghi et al., 2020). Taste buds are regenerated from a stem cell population in the nasal epithelium, the activity of which can be stalled by pro-inflammatory cytokines (Eshraghi et al., 2020). Correspondingly, there are increased serum levels of TNF, IFNG, and IL6 in confirmed COVID-19 patients (Eshraghi et al., 2020). In other diseases where anosmia is seen, it is associated with injury of basal forebrain cholinergic neurons that project to the olfactory bulb, which may drive local microglia to adopt a pro-inflammatory state, contributing to overall neuroinflammation and cell death (Mahalaxmi et al., 2020). Collectively or not, it is thus possible that exacerbated inflammatory responses or CNS hypoxic injuries in COVID-19 patients contribute to these symptoms.

### Encephalopathies

The production of inflammatory mediators as a result of brain viral infection, in particular IFNs (MacMicking, 2012), is associated with encephalitis, a condition characterized by inflammation of the brain parenchyma and neurological dysfunction (Alam et al., 2020). While a few cases of encephalitis associated with COVID-19 were reported, it has been suggested that the symptoms used to diagnose the syndrome could be attributed to other conditions in the patients, such as hypoxia, inflammation, and sedation, which lead to encephalopathies (Garg et al., 2020; Iadecola et al., 2020; Maas, 2020). Common in older, hospitalized COVID-19 patients who present exaggerated systemic inflammation (i.e., cytokine storm), encephalopathy is a pathological process that is characterized by diffuse brain dysfunction (Garg et al., 2020; Helms et al., 2020; Najjar et al., 2020). These patients sometimes present anti-SARS-CoV-2 antibodies and inflammatory markers in the CSF, such as IL6 and IL8 (Alexopoulos et al., 2020; Farhadian et al., 2020; Garg et al., 2020), suggesting encephalopathies could also result from viral infection in the brain [although comprehensive studies are currently lacking].

The presence of anti-viral antibodies and cytokines in the CSF also indicates a potential disruption of the BBB, which may allow for increased inflammatory mediator infiltration into the brain. Enhanced BBB permeability is in line with MRI findings, such as microbleeds, in COVID-19 patients (see section “Microglia Respond to the Systemic Inflammatory Response in COVID-19”) (Fitsiori et al., 2020; Kremer et al., 2020; Radmanesh et al., 2020). Synergistically or not, these stimuli could lead to phenotypic changes of microglia, as supported by post-mortem brain autopsies of COVID-19 patients (see section “Putative Responses of Microglia to SARS-CoV-2 CNS Infection”) (Deigendesch et al., 2020; Matschke et al., 2020). In parallel, a recent report showed enhanced TREM2 protein levels in the CSF of a patient with COVID-19-associated encephalopathy, along with increased protein levels of IL6, IL8, and TNF in both CSF and serum (see section “Microglia Respond to the Systemic Inflammatory Response in COVID-19”) (Pilotto et al., 2020b). The patient robustly responded to steroid treatment, further supporting the argument that the observed encephalopathy was related to a CNS-inflammatory induced event (Pilotto et al., 2020b). While this awaits further research, reactive microglia may contribute to worsening encephalopathy prognosis by ensuing neuroinflammation in COVID-19.

Increased microglial cytokine production, associated or not with BBB breakdown, can also lead to enhanced neuronal excitability and excitotoxic glutamate signaling, possibly provoking seizures, a clinical manifestation of encephalopathies present in COVID-19 patients (Nikbakht et al., 2020). Still, the generation of these seizures may be linked to other microglia-related pathways, including (i) ischemic stroke–which may be due to a microglial response to angiotensin II (see section “Cerebrovascular Disease”), and (ii) mitochondrial dysregulation–which may be caused by an increase in microglial production of ROS (Nikbakht et al., 2020). In mice, excessive extracellular glutamate recruits microglia (Dissing-Olesen et al., 2014; Eyo et al., 2014, 2018), and impairs their phagocytic activity in different epilepsy models (Abiega et al., 2016; Sierra-Torre et al., 2020). Both the microglial recruitment and phagocytosis uncoupling in these models were connected to the disruption of ATP signaling (Eyo et al., 2014, 2018; Abiega et al., 2016; Sierra-Torre et al., 2020), which, as discussed in sections “Microglia Mediate How the CNS Is Affected by SARS-CoV-2 Infection” and “Microglia Could Determine How the CNS Is Affected by COVID-19 Pathology,” can occur during viral and hypoxic injuries. Therefore, during COVID-19, not only can microglia contribute to the onset of seizures, but their reparative activity may also be impaired by the SARS-CoV-2-CNS-related insults.

### Demyelination

In the periphery, there are several inflammatory changes associated with COVID-19, one being the activation of the NLR family pyrin domain containing 3 (NLRP3) inflammasome (Freeman and Swartz, 2020; van den Berg and te Velde, 2020), which potentially contributes to generating the previously discussed cytokine storm (Mehta et al., 2020). In the brain, activation of this inflammasome is a risk factor for developing or worsening multiple sclerosis (Soares et al., 2019) and also Alzheimer’s disease (AD) (see section “Neurodegenerative Diseases”) (Tejera et al., 2019; Heneka et al., 2020), potentially through shifting microglia and macrophages toward pro-inflammatory phenotypes leading to demyelination (Di Stadio et al., 2020). Remarkably, multifocal brain demyelination has been observed in a few cases of COVID-19 (Handa et al., 2020; Zanin et al., 2020). In mice, microglia phagocytose myelin after damage, a crucial step in remyelination (Lampron et al., 2015; Domingues et al., 2020). By contrast, excessive microglial antiviral IFNG response is thought to be the major cause of demyelination in a mouse model of encephalomyelitis (Savarin and Bergmann, 2018). Whether demyelination in COVID-19 is a result of viral or inflammatory-mediated process(es) remains to be investigated, yet, in either case, microglial cytokine production and phagocytosis are likely implicated in this response.

### Cerebrovascular Disease

Although the numbers are increasing rapidly, at least 200 cases of cerebrovascular disease have been reported in association with COVID-19, the majority being caused by ischemic strokes (Ellul et al., 2020; Varatharaj et al., 2020). Accordingly, new and older hypoxic/ischemic injuries were present in numerous brain autopsies of COVID-19 patients (Al-Dalahmah et al., 2020; Kantonen et al., 2020; Matschke et al., 2020; Schaller et al., 2020; Solomon et al., 2020; Weyhern et al., 2020), sometimes accompanied by neuronal loss in the cerebral cortex, hippocampus, and cerebellar Purkinje cell layer (Solomon et al., 2020), as well as brainstem (see section “Microglia Respond to COVID-19-Associated Hypoxia”) (Al-Dalahmah et al., 2020; Weyhern et al., 2020). While most affected patients either already suffer from or belong to high-risk groups for cerebrovascular comorbidities, SARS-CoV-2 seems to increase the risk/rate/prevalence of stroke even in more resilient younger individuals (Ellul et al., 2020). The high incidence of stroke in COVID-19 affected individuals may be partially explained by SARS-CoV-2-associated ECs damage, activating thrombotic and inflammatory pathways, in the CNS and systemically (Varga et al., 2020). Alternatively, SARS-CoV-2 is proposed to, through signaling at the angiotensin type I receptor (AT1R) expressed by microglia, increase the release of pro-inflammatory cytokines (Murta et al., 2020), further inducing vasoconstriction, neuroinflammation, oxidative stress, and cell death (Arroja et al., 2016).

Acute brain hypoxia deriving from systemic COVID-19 and cerebrovascular disease could direct microglial activity toward increased phagocytosis contributing to abnormal neuroplasticity (Serrano-Castro et al., 2020). Reports using pharmacological or genetic depletion of microglia in mice showed a severe reduction of stroke-related injury (Fumagalli et al., 2013; Tang et al., 2014). At the same time, inflammation contributes to injury recovery, helping in immune cell recruitment, scar formation, and astrocytic reactivity (Kim and Cho, 2016). The heterogeneity in microglial phenotypes and subtypes increasingly reported in the literature is proposed as an explanation for the seemingly opposite effects, both neurotoxic and neuroprotective, of these cells observed upon hypoxic/ischemic injury (Stratoulias et al., 2019; Lyu et al., 2020). Despite the lack of comprehensive studies assessing recent territorial ischemic lesions and microglial functions during COVID-19, all autopsied patients with hypoxic injury also showed a history of at least one comorbidity, such as hypertension, diabetes, kidney failure, or obesity among others (Kantonen et al., 2020; Matschke et al., 2020; Schaller et al., 2020; Solomon et al., 2020; Weyhern et al., 2020). Although it is unclear whether these ischemic lesions stem from current COVID-19 infection, comorbidities linked to heightened inflammation, such as pulmonary and cardiac diseases, may indirectly influence microglial reactivity and the release of pro-inflammatory cytokines (Patterson, 2015).

In contexts where microglia are already primed, a secondary immune challenge, such as SARS-CoV-2 infection, may further direct their activity toward neurotoxicity (Serrano-Castro et al., 2020). Primed microglia are thought to abnormally perform their physiological functions, impairing neurogenesis, synaptogenesis, and the structural as well as functional plasticity of brain circuits (Norden et al., 2015; Tay et al., 2017b). Importantly, ensuing neuroinflammation mediated by the activity of microglia, but also astrocytes, is thought to damage the neural tissue and impair synaptic plasticity, critically diminishing cognitive abilities (Lee et al., 2008; Pistell et al., 2010; Davydow et al., 2013; Di Filippo et al., 2013). Thus, in susceptible individuals, an exacerbated inflammatory response of primed microglia to SARS-CoV-2 infection may underlie the onset of disease pathology, including that of psychiatric disorders or neurodegenerative diseases (Norden et al., 2015; Tay et al., 2017b). Furthermore, cerebrovascular events in COVID-19 patients are most prevalent in aging (Ellul et al., 2020; Iadecola et al., 2020), and dysfunctional microglial activity is elevated in older compared to younger individuals (see section “Neurodegenerative Diseases”). Therefore, while hypoxic injuries in COVID-19 patients may be a result of previous chronic conditions, we hypothesize that an already pathological central immune regulation will be further affected by hypoxia-induced upon SARS-CoV-2 infection, leading to exacerbated injury, possibly mediated by primed microglia. As a result, affected individuals would be more at risk to develop neurological symptoms during COVID-19 (Marshall, 2020).

### Neurodegenerative Diseases

Microglial PRR signaling is fundamental to the antiviral IFN response in the brain, particularly at early time points after infection (see section “Putative Responses of Microglia to SARS-CoV-2 CNS Infection”) (Nakagaki et al., 2005; Wheeler et al., 2018; Mangale et al., 2020). IFNs can induce a pro-inflammatory phenotype in microglia, and elevate complement-mediated synaptic pruning in mouse models of AD, the most common cause of dementia worldwide (Hong et al., 2016; Naughton et al., 2020; Roy et al., 2020). Analysis of human AD post-mortem tissue found that plaque-associated IBA1-positive microglia were also immunopositive for the interferon induced transmembrane protein 3 and AXL receptor tyrosine kinase, in line with an IFN pathway activation in AD (Roy et al., 2020). RNA-seq analysis of human AD post-mortem brains also showed a robust correlation between IFN and complement pathways (Roy et al., 2020). A similar phenomenon may occur with SARS-CoV-2 infection, where a viral-induced increase in IFN could lead to increased complement-mediated loss of synapses, resulting in memory or cognitive deficits, across psychiatric, aging, and neurodegenerative conditions.

At the same time, as previously mentioned, NLRP3 inflammasome activation is one of the hallmarks of COVID-19 (Freeman and Swartz, 2020; van den Berg and te Velde, 2020). In humans and mice, the NLRP3 inflammasome plays an important role in AD (Tejera et al., 2019). In mice expressing a human mutation associated with AD, NLRP3 knockout led to enhanced amyloid phagocytosis by microglia, and induced the mRNA expression of anti-inflammatory factors in these cells, including Arginase 1 and *Il4* (Heneka et al., 2013). In the same AD model, NLRP3-knockout also prevented microglial morphological changes after a lipopolysaccharide (LPS) challenge, according to *in vivo* two-photon scanning imaging of the somatosensory system (Tejera et al., 2019). Furthermore, in murine models of tauopathy, NLRP3 accounts for the formation of neurofibrillary tangles, another important component of AD pathology (Ising et al., 2019). Therefore, the increased activation of this complex and the subsequent production of pro-inflammatory factors may induce or worsen neurodegenerative pathology in COVID-19 (Heneka et al., 2020). In addition, in a SARS-CoV-2 infected individual, TREM2 was found to be increased in CSF (Pilotto et al., 2020b). According to human genome-wide association studies, TREM2 variants in microglia are associated with increased risk for AD (Guerreiro et al., 2013) and TREM2 CSF levels strongly associate with CSF levels of tau and phosphorylated tau, important biomarkers for AD (Cruchaga et al., 2013). These findings emphasize the risk for neurodegeneration and correspondent cognitive decline in COVID-19 patients (Heneka et al., 2020).

Parkinson’s disease is often associated with the accumulation of misfolded synuclein alpha leading to a progressive loss of dopaminergic neurons in the *substantia nigra pars compacta* (Lecours et al., 2018). Although there is not yet a reported association with SARS-CoV-2 and parkinsonism, other viruses are associated with a transient form or an increase in the risk of developing PD, partially through microglial reactivity, phagocytosis, and release of pro-inflammatory factors (Sulzer et al., 2020). For example, mouse intranasal infection by influenza viruses (both invading and not invading the CNS) led to an increase in IBA1-positive microglia in the *substantia nigra*, associated either with neuronal transient loss of function or death (Jang et al., 2009; Sadasivan et al., 2015; Tulisiak et al., 2019; Sulzer et al., 2020). Furthermore, during COVID-19, the cytokine storm and the increased AT1R microglial activity may enhance their release of pro-inflammatory cytokines in the CNS (Murta et al., 2020), which may exacerbate protein misfolding and aggregation, mitochondrial dysfunction, and induce a deficiency in autophagy in the brain of SARS-CoV-2 infected individuals (Lippi et al., 2020; Sulzer et al., 2020). This, in turn, may accelerate the development of neurodegenerative disorders, such as PD and AD.

This risk is particularly relevant in older individuals, who have the most severe course of COVID-19, worst post-infection outcomes, and highest mortality rates (Ruan et al., 2020). Importantly, higher ACE2 expression in ECs was found in COVID-19 cases with a history of dementia and hypertension, characterized also by worse outcomes of the infection (Buzhdygan et al., 2020). Indeed, although still understudied, neurological symptoms of COVID-19 are currently shown to be more common in aged patients, along with those with chronic diseases (e.g., diabetes, hypertension) or more severe infections (Mao et al., 2020). Notably, older age is associated with a so-called “inflammaging” phenomenon; an overall decline in immune system efficiency, marked by an elevation of inflammatory markers both in the periphery and CNS (Hammond et al., 2019; Domingues et al., 2020). Older individuals also often display increased numbers of microglia with dystrophic (i.e., smaller cell bodies, seemingly fragmented and tortuous or beaded processes) and reactive morphologies (Streit et al., 2004; Sierra et al., 2007), with the latter partly supporting the concept of microglial priming (Bilbo and Tsang, 2010; Norden et al., 2015; Niraula et al., 2017). Of note, while senescent microglia are frequently used as a synonym for a dystrophic phenotype; it is yet unclear whether this association is valid (Angelova and Brown, 2019). Function-wise, microglia in aged organisms are less motile and less capable of mediating debris clearance (Hefendehl et al., 2014; Safaiyan et al., 2016; Flowers et al., 2017; Marschallinger et al., 2020). When exposed to a challenge, e.g., LPS or laser-induced focal injury, aged microglia, despite being slow to react, can generate an excessive inflammatory response (Sierra et al., 2007; Frank et al., 2010; Damani et al., 2011; Hefendehl et al., 2014; Tejera et al., 2019). For instance, the slower response of aged mouse microglia to a laser-induced focal injury, in the retina and cerebral cortex, was suggested to prevent tissue restoration and contribute to chronic local neuroinflammation (Damani et al., 2011; Hefendehl et al., 2014). After SARS-CoV-2 infection, a dysfunctional response of aged microglia, likely exacerbated by the cytokine storm and hypoxia, may underlie the accelerated cognitive decline observed in aged patients (who did not previously display altered cognition) after a single episode of pneumonia (Shah et al., 2013), a clinical complication that is frequently reported for COVID-19. The molecular basis of this effect could be related to axonal degeneration and myelin loss which were both observed in association with COVID-19 (Handa et al., 2020; Reichard et al., 2020).

Taken together, there is some evidence that microglia are altered upon SARS-CoV-2 infection. Yet, given COVID-19’s novelty, the roles of microglia in the pathogenesis of the disease, particularly in its neurological consequences, are still elusive. Evidence from other viral infections (whether neurotropic or not) points to a potential microglial role in the loss of taste/scent, encephalopathy, cerebrovascular disease, epilepsy, neurodegeneration, and neuropsychiatric concerns (see section “Microglia Respond to Psychosocial Stress”). Furthermore, pre-existing chronic conditions and advanced age may also alter microglia in a way that biases them toward a deleterious contribution to the neurological symptoms. Future work will be essential to clarify the mechanisms underlying the role of microglia in COVID-19’s pathogenesis in the CNS.

## The CNS Is Affected by the Psychosocial Stress Imposed by the COVID-19 Pandemic

### The CNS Is Affected by Psychosocial Stress

On top of the putative neurological burden imposed on infected individuals, the COVID-19 pandemic is associated with increased psychological stress, among both the infected and non-infected populations (Kempuraj et al., 2020; Park et al., 2020). Psychosocial stress is a state of mental, emotional, or physiological strain that results from demanding life circumstances, and persistent maladaptive stress responses are related to various disease conditions including mental health disorders (Tian et al., 2017). To investigate the degree of perceived stress during the pandemic, studies have mainly used self-reporting surveys (Brooks et al., 2020; Ellis et al., 2020). For example, in the United States, electronic health records of 62,354 patients with COVID-19 revealed a significant increase in the prevalence of first diagnosis or relapses of anxiety disorders, insomnia, and dementia, in the 14–90 days following SARS-CoV-2 detection, compared to other health perturbations, such as respiratory and viral infections (Taquet et al., 2020). At the same time, although the percentage of affected individuals varies between reports, survivors of SARS-CoV-1 infection can exhibit impaired memory, sleep disturbance, increased levels of stress, depression, anxiety, and post-traumatic stress disorder (PTSD) symptoms up to 8 years after infection (Wu et al., 2005; Lee et al., 2007; Hong et al., 2009; Lam et al., 2009; Moldofsky and Patcai, 2011). Therefore, psychiatric long-term effects of SARS-CoV-2 infection are also expected.

In the non-infected population, the COVID-19 pandemic imposes a considerable situational stress (Troyer et al., 2020). Fear of being infected, or dying, and uncertainty of the future all contribute to the psychological distress lived by the population (Li et al., 2020b; Mazza et al., 2020; Satici et al., 2020). Social isolation resulting from social distancing and quarantine, changes in lifestyle including sleep, economic recession, financial loss, as well as boredom, misinformation, and overexposure to media coverage of the pandemic can further contribute to this burden (Brooks et al., 2020; Garfin et al., 2020; Kim et al., 2020; Thakur and Jain, 2020). These circumstances along with several others, such as student status, poor self-rated health, higher perceived stress load, worry about family, friends, and other acquaintances suspected of COVID-19, together with less family support, were all associated with an increased risk of developing depressive or anxious symptoms during the pandemic (Vindegaard and Benros, 2020). Accordingly, these stress-contributing circumstances were shown to exacerbate stress-related disorders (Horesh and Brown, 2020) in previous health and economic crises, potentially leading to clinical depression, psychosis, and suicidal tendencies (Chan et al., 2006; Rhodes et al., 2010; Pfefferbaum et al., 2012; Ng et al., 2013).

Certain populations are likely to experience worsened consequences of stressor exposure (Holmes et al., 2020). Susceptible populations include (i) older adults with multiple co-morbidities, (ii) stay-at-home children and women that suffer domestic violence or maltreatment, (iii) people with pre-existing mental health issues or learning difficulties, (iv) health-care workers, as well as (vi) groups struggling socio-economically (Holmes et al., 2020). Regrettably, only a few COVID-19 surveys have yet looked at the non-infected vulnerable groups, such as people with pre-existing mental health conditions. In patients with eating disorders, the symptomatology worsened in 37.5% of responders, whilst 56.2% reported additional anxiety (Fernández-Aranda et al., 2020). In a psychiatric hospital in China, 2,065 outpatients further reported a prevalence of 25.5% anxiety, 16.9% depression, and 26.6% insomnia symptoms, with 20.9% of patients with pre-existing psychiatric disorders presenting a deterioration of their overall mental health state (Zhou et al., 2020). Regarding health-care workers, the majority of papers reveal their increased anxiety, as well as depressive and obsessive-compulsive disorder (OCD) symptoms (Vindegaard and Benros, 2020) [although longitudinal studies are currently lacking in general].

As stress is a prominent risk factor for neurodevelopmental disorders (Bordeleau et al., 2019; Comer et al., 2020), stress loads also need to be investigated in children living during the COVID-19 pandemic. In a survey of 1,054 Canadian adolescents, results indicated increased levels of concern on schooling and peer-relationship while feeling more loneliness and depressive emotions, especially for individuals engaging in longer virtual meetings with friends (Ellis et al., 2020). Although necessary for reducing the spread of the disease, by limiting contact between people, such as between aid specialists and affected populations, there may be reduced access to resources that could alleviate their stress. It is also important to consider children with mood disorders, which can be exacerbated by the pandemic, as well as children with autism spectrum disorders (ASD) or attention-deficit hyperactivity disorder (ADHD) whose lifestyle is disrupted by the change of habits and familiar structures during the pandemic (Jefsen et al., 2020). Overall, children in confinement are subject to reduced physical activity, irregular sleep pattern, and less favorable diets (Carroll et al., 2020; Wang et al., 2020c), which along with their fear-induced chronic stress, loneliness, and viral infection (possibly from COVID-19), represent strong risk factors for developing ASD, ADHD, psychosis, depression, or schizophrenia (Sormunen et al., 2017; Bordeleau et al., 2019; Reeve et al., 2019; Comer et al., 2020). Another risk factor for neurodevelopmental disorders not mentioned yet consists of low family income (Carlsson et al., 2020) for which the number most likely went up during the pandemic with massive job losses (Blustein et al., 2020); together with significant market and bank set-back (Goodell, 2020).

More studies are available looking at mental health distress in the general population. In an online survey that assessed the levels of psychological impact and stress during the initial stage of the COVID-19 outbreak in China, the responses of 1,210 subjects showed that 8.1, 28.8, and 16.5% had moderate to severe stress levels, or anxiety and depression symptoms, respectively (Wang et al., 2020a). The overall mean Impact of Event Scale-Revised (IES-R) score for respondents also indicated the presence of PTSD symptoms (Wang et al., 2020a). In another study with 1,041 Irish respondents, 17.67% met PTSD diagnostic requirements during the lockdown, similar to what the same group found in a parallel study for the United Kingdom (16.79%) (Karatzias et al., 2020). In a large population-based survey conducted in China with almost 53,000 respondents, ∼35% experienced psychological distress (Qiu et al., 2020). Analysis of Weibo (a leading Chinese online social network) posts, based on the approach of Online Ecological Recognition, from almost 18,000 active users, also determined that negative emotions, such as anxiety, depression, and indignation, increased during the pandemic, while positive emotions, like happiness and life satisfaction, significantly decreased (Li et al., 2020a). Moreover, in a Danish study with 2,458 respondents, the World Health Organization Five Well-Being Index (WHO-5) (a psychometrically valid measure of psychological well-being experienced over the past 2 weeks), yielded significantly lower scores during the pandemic compared to results obtained in 2016 (Sønderskov et al., 2020). Likewise, the proportion of respondents for whom assessment for depression should be sought increased compared to the previous survey in 2016 (Sønderskov et al., 2020). Considering the widespread impact of the pandemic, we highlight the need for longitudinal studies investigating stress levels in other cohorts, such as in developing countries or established vulnerable groups, which would allow for a more accurate understanding of stress levels worldwide. In addition, considering the inter-individual response to stress, it would be very important to include additional measures of resilience or vulnerability in these studies.

### Microglia Respond to Psychosocial Stress

It is well established that psychological stress increases the production of pro-inflammatory mediators, peripherally and throughout numerous brain regions implicated in stress-associated neuropsychiatric disorders (Vecchiarelli et al., 2016a; Wohleb and Delpech, 2017; Johnson et al., 2019). Whether it is acute or chronic, stress triggers the sympathetic nervous system, and HPA axis activity, to release catecholamine and glucocorticoids, respectively, from the adrenal glands (Sapolsky et al., 2000). Microglia generally respond to elevated glucocorticoids, cytokines, and catecholamines by altering their density, changing their morphology, and producing pro-inflammatory molecules, i.e., reactivity (Figure 1; Tian et al., 2017). These changes have been linked to the effects of acute stress on the motivational state and cognitive function of the organism, but also to the neurological consequences of prolonged exposure to stress, including atrophy of neuronal dendrites, synaptic loss, and glutamate excitotoxicity (Frank et al., 2019). In line with this, the stress response of microglia is implicated in the pathogenesis of (i) neurodevelopmental conditions, such as ASD and ADHD (Bordeleau et al., 2019), (ii) neuropsychiatric disorders, such as major depressive disorder (MDD), generalized anxiety disorder (GAD), and PTSD (Dantzer et al., 2008; Miller et al., 2009; Haroon et al., 2012; Maes et al., 2012; Leonard, 2014), as well as (iii) neurodegenerative diseases, for instance, AD (Bisht et al., 2018) and PD (Lecours et al., 2018).

#### Stress and Neurodevelopmental Disorders

During critical periods of brain development, exposure to environmental factors (e.g., psychosocial stress) or genetic vulnerabilities, can trigger the onset of neurodevelopmental disorders (Chaste and Leboyer, 2012; Karmiloff-Smith et al., 2012; Wallace, 2016; Carlsson et al., 2020). Correspondingly, rodent models of prenatal maternal stress, induced by sleep deprivation, exposure to bright light, injection of LPS, or of the viral mimic polyinosinic:polycytidylic acid (Poly I:C), produce offspring that have elevated levels of pro-inflammatory markers, as well as impaired microglial density, maturation, and distribution in the postnatal brain, ultimately affecting neurogenesis, synaptic pruning, and brain functional connectivity (Bordeleau et al., 2019). Glucocorticoids and DAMPs released by cellular stress also prime the pro-inflammatory response of microglia *ex vivo* and *in vivo* (Frank et al., 2019). Primed microglia display an increased expression of genes related to phagocytosis, cellular proliferation, and vesicular release, leading to an exacerbated inflammatory response upon exposure to a subsequent challenge. Priming may also occur following acute peripheral inflammation and the increase of circulating cytokines during COVID-19 (see section “Microglia Respond to the Systemic Inflammatory Response in COVID-19”). This could be particularly relevant for children and adolescents infected by SARS-CoV-2 which, despite presumed lower vulnerability to COVID-19 (Lee et al., 2020), are at a critical moment of brain maturation. The same holds for fetuses in pregnant people infected by the virus. A disrupted physiological microglial function may partially explain why maternal stress, and childhood maltreatment, including physical or emotional neglect, are major risk factors for neurodevelopmental disorders and adult psychiatric conditions later in life, such as MDD, PTSD, and GAD (Tay et al., 2017a). Accordingly, impairment of microglial support during critical windows of brain development due to psychosocial stress suggests that infants, children, and adolescents are likely to experience worse neurodevelopmental effects during and after the pandemic (Holmes et al., 2020), possibly potentiated by concomitant SARS-CoV-2 infection (see section “Microglial-Mediated CNS Inflammation may Contribute to the COVID-19 Associated Neurological Manifestations”).

#### Stress and Mental Health in Adulthood and Aging

Psychosocial stress during adulthood does not only increase the risk for MDD and PTSD, but also accelerates cognitive aging, as well as AD and PD progression (Schneiderman et al., 2005; Farrell et al., 2017; Tay et al., 2017a; Benson et al., 2018; Barrero-Castillero et al., 2019; Lähdepuro et al., 2019). Correspondingly, initial reports indicate increased incidence of MDD, anxiety, and PTSD in the general population during the pandemic (Karatzias et al., 2020; Li et al., 2020a; Sønderskov et al., 2020; Wang et al., 2020a). In adult rodents, chronic unpredictable stress (CUS) and social defeat stress models were shown to induce significant increases in microglial density and morphological alterations within areas associated with the stress response and emotion processing (Figure 1; Tynan et al., 2010; Wohleb et al., 2011, 2013; Kopp et al., 2013; Lehmann et al., 2016; Tay et al., 2017a; Nie et al., 2018), although there are many contradictory reports (Tay et al., 2017a). Similar microglial changes were also reported in humans, with post-mortem brains presenting increased microglial density, morphological transformation, and expression of translocator protein 18 kDa (TPSO), which is up-regulated in reactive microglia and astrocytes, across patients with ASD, schizophrenia, MDD, and bipolar disorder (Tay et al., 2017a). Increased IBA1-positive or HLA-DR-positive microglial immunostaining was also encountered in the prefrontal cortex, anterior cingulate cortex, and thalamus of people who have died by suicide (Steiner et al., 2008; Torres-Platas et al., 2014).

The stress-induced increased microglial density and reactivity may account for exacerbated oxidative stress, neuroinflammation, and pathological synaptic remodeling, which are together associated with the development of mental health disorders (Zhao et al., 2015; Milior et al., 2016). For instance, in mice, CUS has been shown to increase the number of phagocytic inclusions containing neuronal elements per IBA1-positive microglial process (Wohleb et al., 2011, 2013), which is suggestive of augmented synaptic pruning. Considering that synaptic loss is the best pathological correlate of cognitive decline in MDD, schizophrenia, aging, and neurodegenerative diseases (Terry et al., 1991; Tay et al., 2017a), modulating the synaptic remodeling executed by microglia in response to stress could allow to mitigate the detrimental effects of psychosocial stress. To do so, one alternative could be to target fractalkine signaling between neurons and microglia, via CX3CR1, since several works suggest that CX3CR1-deficient mice are resistant to the deleterious effects of chronic stress exposure (Tay et al., 2017a). Specifically, in adult mice, CX3CR1-deficiency prevented the effects of CUS on neuronal plasticity in the hippocampus CA1 region, as well as the emergence of depressive-like behavior (Milior et al., 2016). These mice were also found to be resistant to stress-induced microglial hyper-ramification in the dentate gyrus, and depression-like behavior under the forced swim paradigm (Hellwig et al., 2016). Of note, disrupting this pathway appears detrimental during pre- and postnatal development (Paolicelli et al., 2011; Squarzoni et al., 2014; Zhan et al., 2014), and during viral and hypoxic injuries (see sections “Putative Responses of Microglia to SARS-CoV-2 CNS Infection” and “Microglia Respond to COVID-19-Associated Hypoxia”), thus, more research is necessary to determine the feasibility of this approach.

Stress resulting from the pandemic (and viral infection) could also enhance the activity or development of microglia associated with higher synaptic pruning activity, for instance, “dark microglia,” which could be targeted specifically (Bisht et al., 2016; El Hajj et al., 2019; Savage et al., 2020; St-Pierre et al., 2020). There are also stress-induced microglial-mediated changes in neurotransmitter synthesis, altogether allowing for a neural environment where neuroinflammation and alterations in neuronal activity contribute to the development of mental health diseases. In response to inflammatory stimuli or stress, in microglia, the tryptophan metabolite kynurenine [from the indoleamine 2,3-dioxygenase (IDO) pathway] is used to form quinolinic acid, a glutamate ionotropic receptor agonist [N-methyl-D-aspartate receptor (NMDAR)] (Capuron et al., 2002; Schwarcz et al., 2012; Vecchiarelli et al., 2016b). Remarkably, there is an increase in the expression of genes involved in tryptophan metabolism with COVID-19 (Gardinassi et al., 2020), which could lead to glutamate excitotoxicity and divert tryptophan away from serotonin synthesis toward kynurenine metabolism (Capuron et al., 2002; Schwarcz et al., 2012; Vecchiarelli et al., 2016b), associated with anxiety, mood disorders, psychosis, and cognitive decline. This is likely contributed to by the systemic inflammation associated with COVID-19.

The effects of stress on microglia depend on the type, duration, and frequency of the exposure (Yirmiya and Goshen, 2011; Calcia et al., 2016). The myriad of psychosocial stressors during the pandemic will likely have diverse effects on microglia, but we hypothesize that vulnerable groups carrying an already primed immune response, for instance, due to aging, chronic disease, or a history of mental distress, will experience more pro-inflammatory outcomes, in turn, resulting in an increased risk for psychiatric disorders. We highlight the potential threat of a global mental health crisis affecting these populations and the urgent need for policies that provide targeted care, including access to the COVID-19 vaccine.

## Discussion and Conclusion

The COVID-19 pandemic possibly affects the CNS through viral infection, hypoxic-injuries, and increased cytokine circulation in SARS-CoV-2 infected individuals, but also by psychosocial stress, in both non-infected and infected populations. Microglia, crucial to physiological and immune functions of the brain, respond to these insults in diverse ways. In a model of murine coronavirus infection (JMHV), ablation of these cells with CSFR1 inhibition promoted deleterious outcomes, including increased animal mortality, viral replication, T cell infiltration, and demyelination (Mangale et al., 2020). Microglial depletion also exacerbates stroke-injury and reduces neurogenesis (Lalancette-Hébert et al., 2007; Szalay et al., 2016). This indicates, at least in some models of CNS viral infection and CNS injury, that microglia (amongst other cells) serve a protective role, and that their reduced presence allows for even more deleterious effects. However, in the context of a cytokine storm or after exposure to chronic psychosocial stress, microglia can become altered in their function and then increase the release of inflammatory mediators, generating pathogenic effects associated with neurological and psychiatric conditions. These combined findings highlight a need to understand how microglia (and their different subtypes) may switch temporally from contextually beneficial states to harmful ones. This knowledge would be valuable to mitigate harmful CNS outcomes of COVID-19 pandemic, and possibly its long-term consequences on neurodevelopmental, psychiatric, and neurodegenerative conditions. Alternatively, although this awaits further research, in critical groups, for instance, aged individuals or cohorts with pre-existing conditions, modulating the pro-inflammatory activity of microglia upon SARS-CoV-2 detection, via minocycline (Oliveira et al., 2020), for example, may help avoid unwanted neurological outcomes. The same holds for individuals with a history of mental health disorders, for which the reduction of dysfunctional microglial synaptic remodeling may prevent the worsening of mental distress associated with the pandemic.

## Author Contributions

EGA and M-ÈT conceptualized the manuscript topic with input from all authors. MC created the figure included in the manuscript with the help of EGA and EŠ. All authors contributed to the writing and editing of the manuscript.

## Conflict of Interest

The authors declare that the research was conducted in the absence of any commercial or financial relationships that could be construed as a potential conflict of interest.
